# Supplementary material for: DMRIntTk: Integrating different DMR sets based on density peak clustering
Source: PLoS One. 2024 Dec 23;19(12):e0315920. doi: 10.1371/journal.pone.0315920 (PMC11666043; doi:10.1371/journal.pone.0315920)
Supplement: Attachment — Submitted filename: Response letter.docx [file pone.0315920.s001.docx]

Authors' Responses to the Editor's Comments

On the paper

**“DMRIntTk: integrating different DMR sets based on density peak clustering”**

By Wenjin Zhang, Wenlong Jie, Wanxin Cui, Guihua Duan, You Zou, Xiaoqing Peng

Dear the Editor,

We would like to thank you for your positive comments and recommendation. We also thank the reviewers for careful reading, helpful comments, and constructive suggestions, which have significantly improved the presentation of our revised paper.

This submission has been revised after carefully reading all comments. In the following pages, we give our point-wise responses to the review comments. We have copied the original comments from the reviewers, and made them italic. Our responses are given in roman.

We wish our revised manuscript and responses would satisfy you.

Xiaoqing Peng and You Zou

School of Life Sciences,

Central South University

Changsha, Hunan, P.R. China

Email: xqpeng@csu.edu.cn

**Response to Reviewer 1**

**Reviewer’s comments**

*The manuscript 'DMRIntTk: integrating different DMR sets based on density peak clustering' by Zhang et al. developed a new toolkit - DRMIntTk - which integrates DMR regions from multiple preexisting software packages to identify DMR regions with high significance in multiple software analysis, but not necessarily only the DRMs with the highest modifications. DRM data from the Illumina 450K chip and submitted previously to NCBI was used for the analysis. The DMRIntTk output (ie Fig 2) does show identification of midrange methylation peaks that are missed by the separate programs based on default settings. Additional tissue studies show a similar enhancement of potential areas of interest using DMRIntTk vs. other software. The author's then use the DRMIntTk data and GO enrichment analysis to show that regions of interest contain genes potentially being influenced via methylation.*

**Authors' Response**

Thanks for your positive comments.

**Reviewer’s comments**

*While the GO enrichment analysis identifies methylated regions of genes that make 'biological sense' for the different tissue analysis done, it is an observation/correlation, not a conclusion of biological function.*

**Authors' Response**

Thank you for your comments. In our revised manuscript, we have performed KEGG analysis on the integrated DMR set between PCa tissues and adjacent normal tissues, as shown in Figure 8(b). The KEGG enrichment analysis identified “neuroactive ligand-receptor interaction” as the most significant pathway associated with the integrated DMR set from PCa. Evidence from a recent study [1] indicates a strong link between this pathway and PCa.

The additional analysis will help validate the biological functions of the DMR sets of interest by providing a broader context of pathway involvement and functional significance. By incorporating KEGG pathways, we aim to strengthen the evidence for the biological relevance of the identified DMRs, offering a more comprehensive understanding of their potential roles in the pathological mechanisms of the diseases studied.

**Fig 8. Functional enrichment analysis of the integrated DMR set on PCa versus normal.** (a) GO enrichment analysis, (b) KEGG pathway enrichment analysis.

**Response to Reviewer 2**

**Reviewer’s comments**

*In this manuscript (PONE-D-24-31358) entitled "DMRIntTk: integrating different DMR sets based on density peak clustering" submitted to PLOS ONE, the authors develop DMRIntTk, a pipeline for the integration of DMRs predicted by other tools. The pipeline was implemented in a four-step method including, genome segmentation, construction of a reliability matrix, bins weighting and DMRs integration. As prove of concept, the authors analyzed different datasets with low, medium and large methylation differences.*

*The main advantages of DMRIntTk over the other methods are the increased number DMRs with higher methylation differences and the increased proportion of DMRs. Finally, the results of GO enrichment analysis involve terms associated to the samples being analyzed.*

**Authors' Response**

*Thank you for your constructive and supportive comments.*

**Reviewer’s comments**

*1. Even though the manuscript is well written and clear, my main concern comes from the “biological advantages of the method”. The pipeline seems to have good performance increasing the proportion of DMRs with higher differences, but the question is, it was any important gene/GO term that was not present in the original DMRs? It will be very interesting to see the differences in GO terms and DMGs. On the other hand, to use the pipeline, the users would have to run some or all the other tools before, so it must be clear that the biological results of DMRIntTk are superior to any of the other individual analyses. If the main propose of the pipeline is to find new biological insight that could not be found with other individual tool the authors should present it on the paper. To see a real advantage of DMRIntTk over other methods, more results focusing on the biological aspect are necessary. The authors should amend these issues before publications.*

**Authors' Response**

Thank you for your suggestive comment. In the revised manuscript, we have added the Section 3.6.3, “Comparison of KEGG pathway enrichment analysis between the integrated DMR set and the original DMR sets”, to address your concern regarding the biological advantages of DMRIntTk. In this section, we have included additional comparison analyses that highlight important pathways which are uniquely identified by DMRIntTk pipeline. By comparing the integrated DMR set between PCa tissues and adjacent normal tissues with the original DMR sets, the number of shared significant pathways between the integrated DMR set and each original DMR set is shown in Figure 10.

**Fig 10. Upset plot of shared significant pathways between the integrated DMR set and each original DMR set.**

The upset plot illustrates that, several pathways that were significantly enriched in the integrated DMR set but not in all or the majority of the original DMR sets, such as asthma, pancreatic secretion and protein digestion and absorption. These pathways have been well-documented in the literature as being associated with PCa [2-5], which emphasizes the biological relevance and potential insights gained from utilizing the DMRIntTk pipeline. This comparison underscores the unique biological insights provided by our method, which may not be readily apparent when using individual methods.

**Reviewer’s comments**

*See also my comments below:*

*2. Introduction:*

*Please include in the introduction a clear definition of DMRs and the different methods available to detect CpG such as sequencing, array, etc. A paragraph highlighting the main advantage and disadvantage (including references) of the tools would help to understand the strength of the DMRIntTk.*

**Authors' Response**

Thank you for your comment. In the revised manuscript, we have modified the introduction to include a clear definition of differentially methylated regions (DMRs) and an overview of various methods available for detecting CpG methylation. We have also added the discussion about the advantages and limitations of several widely used tools for DMR detection, including ProbeLasso, DMRcate, combp, bumphunter, and seqlm, supported by relevant references. We believe this addition provides a clearer context for understanding the strengths of DMRIntTk in comparison to other available tools.

**Reviewer’s comments**

*Methods:*

*3. Explain better how the bins are obtained. Could the bins introduce a vias in the analysis?*

**Authors' Response**

Thank you for your comments. In the revised manuscript, we have provided *more detail on how the bins are obtained.* First, adjacent probes with a genomic distance of less than 500 bp were clustered together to form fragments. Then, a 500 bp sliding window was applied to segment fragments larger than 500 bp, dividing them into continuous, non-overlapping bins. The bins provide a consensus basis for analyzing the DMR sets predicted by different methods. We do not anticipate that the bins introduce any significant bias or variance in the analysis, as the bins are defined in a way that reflects the inherent probe distribution, and the window size is designed to balance resolution and genomic coverage.

**Reviewer’s comments**

*4.* *Seems that the algorithm was implemented to filter DMRs with low differences. Some levels of low differences are always expected.*

**Authors' Response**

Thank you for your comments. The algorithm is not implemented to filter DMRs with low differences. Since the bins are based on both their methylation differences and the reliability scores of the covering DMR set, the bins with small methylation differences but high reliability scores will still be retained.

**Reviewer’s comments**

*5. The different segmentation of the genome could impact on the number of DMRs, was this effect was evaluated?*

**Authors' Response**

Thank you for your suggestive comment. In our revised manuscript, to evaluate the impact of different genome segmentation strategies on the integrated DMRs, we have run DMRIntTk with different genome segmentation strategies and compared the integrated DMR sets. Given a length of *k* bp, first, adjacent probes with a genomic distance of less than *k* bp were clustered together to form fragments. Then, a *k* bp sliding window was applied to segment fragments larger than *k* bp, dividing them into continuous, non-overlapping bins. Specifically, we have applied the lengths of 50 bp, 200 bp, 500 bp, and 800 bp.

As shown in Figure 11, the results indicate that the distributions of methylation differences of integrated DMR sets generated by different segmentation strategies are generally similar. When the methylation difference threshold *t* falls within the range of [0.1, 0.3], particularly in the range of [0.1, 0.2], we observed that the proportion of DMRs in the integrated set was significantly higher than in the original DMR sets.

Notably, the 50 bp and 800 bp segmentation strategies exhibited a relatively smaller proportion of DMRs with methylation differences greater than 0.2. This could be attributed to the fact that shorter lengths (e.g., 50 bp) may omit informative probes, while longer lengths (e.g., 800 bp) may include regions with minimal methylation differences. Despite these differences, the overall trends were consistent across the different segmentation strategies.

In conclusion, different lengths do have some impact on the integrated DMRs. Based on the comparison of different integrated DMR sets generated by DMRIntTk with different segmentation strategies, we suggest that the length of 500 bp, used in this study, provides a suitable and balanced approach for genome segmentation.

**Fig 11.** **Methylation difference distributions of different integrated DMR sets generated by DMRIntTk with different segmentation strategies.**

**Reviewer’s comments**

*6. Please follow the pipeline in the methods sections, move the “Segment the genome and weight the bins” before “Construct reliability matrix”.*

**Authors' Response**

Thank you for your suggestion. In the revised manuscript, we have made some revision to make the pipeline more clearly. In our study, the steps “Construct reliability matrix” and “Segment the genome” can be performed parallelly, and the bin weighting is computed based on these both steps. Therefore, in the manuscript, Figure 1 has been modified by exchanging the modules of "Construct reliability matrix" and "Segment the genome". In the Methods section, we have combined the "Segment the genome" and "Weight the bins" steps into a single description.

**Reviewer’s comments**

*7. Explain clearly the input and output of the pipeline.*

**Authors' Response**

Thank you for your helpful suggestion. In the revised manuscript, we have added a detailed explanation of the input and output of the pipeline. The inputs include a pre-processed methylation array data, the group information of samples, along with several specified parameters. These parameters include one or more desired DMR prediction methods, the array type, the minimum number of probes, and preferences related to DMR characteristics (such as a preference for longer regions or regions with higher probe density). The output of DMRIntTk is a file containing the set of integrated DMRs.

**Reviewer’s comments**

*8. Results:*

*In this section, the authors focus on the ratio of DMRs differences and the overlapping. Please, include the same comparative analysis (i.e, individual tool VS DMRIntTk) with the genes and terms in the different datasets analyzed.*

**Authors' Response**

Thank you for your suggestive comment. In our revised manuscript, we have added Section 3.6.3, titled "Comparison of KEGG pathway enrichment analysis between the integrated DMR set and the original DMR sets." In this section, we provide a comparative analysis of KEGG pathway enrichment between the integrated DMR set and each individual DMR set. This analysis can highlight the important pathways that are uniquely identified by DMRIntTk. Specifically, we have compared the integrated DMR set from prostate cancer (PCa) tissues and adjacent normal tissues with the original DMR sets, and the number of shared significant pathways between the integrated DMR set and each of the original DMR sets is shown in Figure 10.

**Reviewer’s comments**

*9. How the DMGs were analyzed? Usually, the gene body methylation is separated from the upstream and downstream methylation.*

**Authors' Response**

Thank you for your suggestive comment. In this manuscript, to extract DMGs, firstly the annotations of the Illumina methylation array is used to extract the probes contained in the DMR, and then the DMR located genes (DMGs) are extracted based on the annotation of probes. As we known, a large portion of probes in Illumina methylation arrays (e.g., Infinium 450K or 850K BeadChip) are located in promoter regions, especially near the transcription start site (TSS). In Section 3.6, we have elaborated on how the DMGs are obtained based on DMRs, specifying the location of our DMRs in the gene. Next, the GO and KEGG enrichment analysis is performed on these DMGs by clusterProfiler R package[6].

**Reviewer’s comments**

*10. Discussion:*

*Discuss what it was stated in the introduction, was DMRIntTk better at finding some type of DMR?*

**Authors' Response**

Thank you for your insightful comment. In the revised manuscript, we have discussed the advantages of DMRIntTk in detail in the Conclusions and Discussion section.

It excels at integrating DMRs identified by different methods, taking into account both the reliability of the detection methods and the magnitude of DNA methylation differences. By weighting DMRs according to these factors, DMRIntTk is able to prioritize and identify more reliable DMRs that exhibit larger methylation differences, while filtering out DMRs with smaller weight (i.e., those with lower methylation differences or less reliable detection).

**Reviewer’s comments**

*11. It could be beneficial if the tool could read directly datasets obtained by other methods such as EMseq, bisulfite, long read sequencing etc.*

**Authors' Response**

Thank you for your suggestive comment. In the current version, DMRIntTk can be applied for array data. We plan to enhance the tool in the future to incorporate these additional datasets.

**Response to Reviewer 3**

**Reviewer’s comments**

*In this manuscript, the authors present the results obtained from the integration of different DMR sets and the development a specific toolkit for evaluating the reliability of different DRM sets with the general aim to detect and enhance the proportion of DMR regions with higher levels of methylation.*

*In general, the paper appears clear in writing. The authors well summarized and presented the importance of their purpose and their case of study. The followed experimental pipeline appears correct and the obtained results have been described quite clearly.*

**Authors' Response**

Thanks for your positive comments.

**Reviewer’s comments**

*1. However, in Introduction section, there is a little emphasis on the description of the biological process of DNA methylation as epigenetic aspect and the discussion of the results not appears sufficiently clear to define if this new toolkit could be applied also for other cases of study (i.e in plant systems, where the methylation levels related to asymmetric contexts CNN or CNG are frequent). In the absence of a comprehensive description of the biology the final discussion section appears uninvolving. For this reason, it becomes difficult to read and unclear at some points. Formatting the manuscript differently would be important to clearly define some points, for example with a more exhaustive description of the biology behind this system followed by a short section on the technique to take advantage of the biology.*

**Authors' Response**

Thank you for your comments. In the revised manuscript, we have provided a more comprehensive discussion of DNA methylation biology in the context of both animals and plants. We have included a detailed explanation of DNA methylation's role in gene expression, transposon mobility, and genome stability, emphasizing the differences between symmetric CpG methylation in mammals and asymmetric CHH/CHG methylation in plants.

Furthermore, we have clarified the potential applicability of DMRIntTk to plant systems, including planned modifications to adapt the toolkit for plant-specific methylation patterns.

**Reviewer’s comments**

*Although the topic is of extreme interest, in my opinion, there are some issues with the focus of the work Therefore, I have described a few major and minor concerns, based on my skills. I had limited my report to some aspects highlighting some issues.*

*Minor comments:*

*2. Line 58:*

*“Let mmax denote the maximum methylation difference” in my opinion it should be mentioned as it comes measured this value, the authors do it later in the discussion but it should be anticipated.*

**Authors' Response**

Thank you for your comments. We have revised the text to clarify how the maximum methylation difference, denoted as m_max_, is measured. The updated section now anticipates this information before the discussion.

**Reviewer’s comments**

*3. Equation 1:*

*Some terms could be explained better (like Ni to the fourth) but maybe I don't have them suitable skills for judging.*

**Authors' Response**

Thank you for your comment. In the revised manuscript, the descriptions of the terms including the *N_i_^4^* term have been provided a clearer explanation. The intention behind raising *N_i_* to the fourth power is to emphasize the contribution of intervals with higher probe densities, thus improving the overall reliability of the *DMRscore*. We hope the revised explanation now offers a more comprehensive understanding of the formula.

**Reviewer’s comments**

*4. Figure 1:*

*In Figure 1 the expression Diff(bin) is used to indicate the absolute value of the difference of methylation associated with a Bi segment; however, the expression is always used in the text and in the formulas Dif(Bi). In my opinion, if the authors are the same thing, the figure should be corrected, otherwise the figure should be clarified difference to avoid confusion.*

**Authors' Response**

Thank you for your suggestive comment. In the revised manuscript, we have corrected the figure to ensure that the expression “Dif(bin)” is consistently used throughout the figure, text and formulas. This should eliminate any confusion regarding the notation.

**Reviewer’s comments**

*5. Line 365:*

*A dataset SE50192 is mentioned, it is actually GSE50192, correct the ID.*

**Authors' Response**

Thank you for your comment. In the revised manuscript, we have amended the text from “SE50192” to “GSE50192” to ensure accuracy.

**Reviewer’s comments**

*6.* *Line 367:*

*I was unable to access data from this dataset: Religious Orders Study and Memory and Aging Project (ROSMAP), I don't understand if it's paid or if the type of searching is wrong.*

**Authors' Response**

Thank you for your comment. We apologize for the confusion in our previous description. To clarify, the Religious Orders Study and Memory and Aging Project (ROSMAP) is a dataset that includes various types of omics data in AD. The ID syn3219045 refers to the super-series dataset, while the AD epigenetic data we utilized is the sub-series dataset with ID syn3157275. Additionally, access to ROSMAP data requires the necessary permissions from Synapse and the AD Portal. In the revised manuscript, we have changed to the correct ID and clarified the access requirements for this dataset.

**Reviewer’s comments**

*7.* *Line 374:*

*Typo “cluatering” fix clustering*

**Authors' Response**

Thank you for your point. We have corrected “cluatering” to “clustering” in the revised manuscript.

**Reviewer’s comments**

*8. The part about overlap analysis should be explained better: from the text it is not clear which one is comparing one method at a time with theirs, however, it is clear in the figures.*

**Authors' Response**

Thank you for your comments. In the revised manuscript, we have ensured that the comparison of our method with others is articulated more effectively, so readers can easily follow the analysis alongside the figures.

**Reviewer’s comments**

9. Moreover, there is a little of confusion with the terms individual DMR set/original DMR set.

**Authors' Response**

Thank you for your comment. The terms "individual DMR set" and "original DMR set" both refer to the DMR set predicted by a DMR prediction method. However, the individual DMR set is mostly used when comparing the integrated DMR set with the DMR set predicted by a single method, which emphasizes the comprehensiveness of the integrated DMR set, and the original DMR set is mostly used to emphasize the differences between the integrated DMR set and the original DMR set.

**Reviewer’s comments**

10. As for the validation part of the results, in the end they do a GO for each comparison.

My personal question is related to the possibility to apply an alternative validation analysis to get more evidence.

**Authors' Response**

Thank you for your suggestive comment. In the revised manuscript, we have included the KEGG pathway enrichment analysis to provide further evidence of the biological significance of the DMRIntTk integrated DMR set. Additionally, we have added the section 3.6.3, “Comparison of KEGG pathway enrichment analysis between the integrated DMR set and the original DMR sets”, in which we have performed a series of comparative analyses comparing the significantly enriched pathways in the integrated DMR set with those in the original DMR sets to demonstrate the superiority of DMRIntTk over other methods. We believe this will strengthen our findings and offer a more robust validation.

**Reviewer’s comments**

*11. Finally, I personally believe that it is necessary to better explain and evaluate whether and to what extent a similar tool can be used transversally in other biological systems and in other contexts of asymmetric methylation.*

Authors' Response

**Authors' Response**

Thank you for your constructive and supportive comments. In the revised manuscript, we have added a discussion regarding the potential applicability of DMRIntTk to other biological systems and contexts of asymmetric methylation, especially, plant systems. To adapt DMRIntTk for plant systems, we have outlined our planned modifications, such as using adaptive-length bins for genome segmentation instead of the fixed-length bins used for human data and incorporating genomic annotations.

[1] Gallon J, Rodriguez-Calero A, et al. DNA Methylation Landscapes of Prostate Cancer Brain Metastasis Are Shaped by Early Driver Genetic Alterations. Cancer Res. 2023;83(8):1203–1213

[2] Su YL, Chou CL, et al. sthma and Risk of Prostate Cancer: A Population-Based Case-Cohort Study in Taiwan. Medicine (Baltimore). 2015;94(36):e1371.

[3] Severi G, Baglietto L, et al. Asthma, asthma medications, and prostate cancer risk. Cancer Epidemiol Biomarkers Prev. 2010;19(9):2318–2324.

[4] Ahearn TU, Peisch S, et al. Expression of IGF/insulin receptor in prostate cancer tissue and progression to lethal disease. Carcinogenesis. 2018;39(12):1431–1437.

[5] Raina K, Peisch S, Lu J. PROTAC-induced BET protein degradation as a therapy for castration-resistant prostate cancer. Carcinogenesis. 2016;113(26):7124–7129.

[6] Yu G, Wang LG, et al. clusterProfiler: an R package for comparing biological themes among gene clusters. OMICS. 2012;16(5):284–287.

**Editor's Comments to Author:**

*Editor's Comments to Author:*

*Editor*

*Comments to the Author:*

*(There are no comments.)*
